# The all-on-four treatment concept: Systematic review

**DOI:** 10.4317/jced.53613

**Published:** 2017-03-01

**Authors:** David Soto-Penaloza, Regino Zaragozí-Alonso, María Penarrocha-Diago, Miguel Penarrocha-Diago

**Affiliations:** 1 Collaborating Lecturer, Master in Oral Surgery and Implant Dentistry, Department of Stomatology, Faculty of Medicine and Dentistry, University of Valencia, Spain; 2 Dentist, Department of Stomatology, Faculty of Medicine and Dentistry, University of Valencia, Spain; 3 Assistant Professor of Oral Surgery, Stomatology Department, Faculty of Medicine and Dentistry, University of Valencia, Spain; 4 Professor and Chairman of Oral Surgery, Stomatology Department, Faculty of Medicine and Dentistry, University of Valencia, Spain

## Abstract

**Objectives:**

To systematically review the literature on the “all-on-four” treatment concept regarding its indications, surgical procedures, prosthetic protocols and technical and biological complications after at least three years in function.

**Study Design:**

The three major electronic databases were screened: MEDLINE (via PubMed), EMBASE, and the Cochrane Library of the Cochrane Collaboration (CENTRAL). In addition, electronic screening was made of the ‘grey literature’ using the System for Information on Grey Literature in Europe - Open Grey, covering the period from January 2005 up to and including April 2016.

**Results:**

A total of 728 articles were obtained from the initial screening process. Of these articles, 24 fulfilled the inclusion criteria. Methodological quality assessment showed sample size calculation to be reported by only one study, and follow-up did not include a large number of participants - a fact that may introduce bias and lead to misleading interpretations of the study results.

**Conclusions:**

The all-on-four treatment concept offers a predictable way to treat the atrophic jaw in patients that do not prefer regenerative procedures, which increase morbidity and the treatment fees. The results obtained indicate a survival rate for more than 24 months of 99.8%. However, current evidence is limited due the scarcity of information referred to methodological quality, a lack of adequate follow-up, and sample attrition. Biological complications (e.g., peri-implantitis) are reported in few patients after a mean follow-up of two years. Adequate definition of the success / survival criteria is thus necessary, due the high prevalence of peri-implant diseases.

** Key words:**All-on-four, all-on-4, tilted implants, dental prostheses, immediate loading.

## Introduction

The “all-on-four” treatment concept was developed to maximize the use of available remnant bone in atrophic jaws, allowing immediate function and avoiding regenerative procedures that increase the treatment costs and patient morbidity, as well as the complications inherent to these procedures (1). The protocol uses four implants in the anterior part of complete edentulous jaws to support a provisional, fixed and immediately loaded prosthesis. The two most anterior implants are placed axially, whereas the two posterior implants are placed distally and angled to minimize the cantilever length, and to allow the application of prostheses with up to 12 teeth, thereby enhancing masticatory efficiency (2,3).

The original Brånemark surgical-prosthetic protocol advocated the placement of four implant fixtures for the restoration of a resorbed mandible and 6 implant fixtures on mandibles that demonstrated minimal to moderate resorption (4), as a prelude to the subsequent tendencies (2).

Immediate loading procedures for edentulous jaws have become widely popular among clinicians as well as among patients (5,6). High survival rates and a low incidence of complications demonstrate the predictability of implant treatment, regardless of the loading regimen involved (7,8). The challenge today is not to prove functionality but rather to develop simple and cost-effective protocols.

This all-on-four concept has been described by several studies and clinical reports, summarized in a previous review (9). However, at that time the main descriptions were limited to survival rates, implant failures and technical complications, with little emphasis being placed on biological complications such as peri-implant diseases, which are currently considered to be very frequent (10,11).

There are gaps in the literature related mainly to the therapeutic indications, since no consensus has been established regarding surgical procedures and prosthetic protocols. The aim of this systematic review was to summarize and update the all-on-four treatment concept, as well as the surgical and prosthetic topics based on clinical studies offering results after a follow-up of at least 36 months.

## Material and Methods

The present systematic review was conducted based on the guidelines of Transparent Reporting of Systematic Reviews and Meta-Analyses – PRISMA Statement (Moher *et al.* 2009) (12).

- Focus question 

The focus question was established according to an adaptation of the PICO structured question, in this case applying a PEO (population, exposition, outcome) format, and considering the importance of including observational studies without a comparative group, such as single cohort studies. This approach is adequate for performing qualitative systematic reviews in health interventions. The question format was established as follows: “In edentulous patients or with severely resorbed jaws that receive dental implants for immediate full-arch implant-supported restorations following the all-on-four concept in the mandible or maxilla, what are the most frequent clinical indications, surgical procedures, prosthetic protocols and complications?”

P (population): Edentulous patients with atrophic maxilla.

E (exposition): Placement of four implants with immediate loading of a prosthesis following the all-on-four concept.

O (outcome):

O 1: Treatment indications, surgical procedures, prosthetic protocols (loading time, prosthetic material, abutment, type of fixation, occlusal control).

O 2: Technical complications (prosthesis fracture, abutment fracture, screw fracture or losses).

O 3: Biological complications (mucositis, peri-implantitis, implant failure).

- Information sources and data extraction

Electronic and manual literature searches were conducted by two independent reviewers (DSP, MPD), while another two reviewers independently extracted the data from studies (DSP, RZA). Publications that did not meet the inclusion criteria were excluded. In the case of disagreement, consensus was reached through discussion with a fourth reviewer (MPD).

- Screening process

The three major electronic databases were screened: MEDLINE (via PubMed), EMBASE, and the Cochrane Library of the Cochrane Collaboration (CENTRAL). In addition, electronic screening was made of the ‘grey literature’ at the System for Information on Grey Literature in Europe - Open Grey (http://www.opengrey.eu/), as recommended by the AMSTAR (quality assessment of systematic reviews) guidelines (13). The search contemplated papers published without language restrictions from January 2005 up to and including April 2016. The search strategy included a combination of the controlled terms (MeSH and EMTREE), and keywords were used whenever possible in an attempt to obtain the best search results. In addition, other terms not indexed were used. As a complement, a manual search of main primary source related topics was performed, and the reference lists of definitely included articles were consulted to find possible eligible studies. The following search strategy was carried out.

PEO search: ((((edentulous atrophic maxilla OR edentulous OR alveolar ridge atrophy OR atrophy maxilla OR atrophic maxilla OR atrophic mandible OR atrophied maxilla OR “Jaw, Edentulous”[Mesh] OR “Alveolar Bone Loss”[Mesh] OR “Mouth, Edentulous”[Mesh] OR edentulous mandible OR edentulous jaw))) AND (((fixed implant prosthesis OR immediate function OR full-arch fixed dental prostheses OR cross-arch fixed dental prosthesis OR screw fixed prostheses OR “Dental Implant-Abutment Design”[Mesh] OR inclined abutment OR angulated abutment OR straight abutment OR All-on-4 (R) OR all-on-4 concept OR all-on-4 surgery OR all-on-4 OR all-on-four OR all on four OR all on 4 OR four dental implants OR 4 dental implants OR dental AND (tilted implants OR axial implants OR distal tilted implants OR distal angulated implants OR distal inclined implants OR distal angle implants OR axial dental implants OR axially implants))) OR ((all-on-4 AND (“Immediate Dental Implant Loading”[Mesh] OR “Dental Implants”[Mesh] OR immediate loading OR early loading OR cad-cam OR cad/cam technology OR nobelguide OR guided surgery OR guided implant placement OR flapless implant surgery OR post-extractive implants))))) AND ((“Immediate Dental Implant Loading”[Mesh] OR “Dental Implants”[Mesh] OR loading protocol OR immediate loading OR early loading OR surgical protocol OR surgical procedure OR post-extractive implants OR surgical complication OR “Postoperative Complications”[Mesh] OR biological complication OR biological complications OR “Peri-Implantitis”[Mesh] OR peri implantitis OR peri-implant mucositis OR periimplant mucositis OR “Stomatitis”[Mesh] OR “Dental Restoration Failure”[Mesh] OR technical complications OR technical complications OR technical complication OR abutment fracture OR dental prostheses fracture OR acrylic fracture OR screw loss OR screw fractures OR dental implant failure OR “Computer-Aided Design”[Mesh] OR cad/cam technique OR cad-cam OR nobelguide OR “Surgery, Computer-Assisted”[Mesh] OR guided surgery OR guided surgery OR guided implant placement)).

- Risk of bias and quality assessment

Two reviewers (DSP and RZA) designed and assessed the proposal for the present project to ensure compliance with the PRISMA guideline in order to avoid risk of bias and provide a high level of evidence. PRISMA consists of a 27-item checklist and a four-phase flow diagram (12). Two independent reviewers (DSP and RZA) evaluated all the included articles.

The methodological quality of observational studies was assessed with the Newcastle-Ottawa Scale (14), and the Cochrane Collaboration tool for assessing the risk of bias was employed for the assessment of randomized controlled trials (RCTs).

For each aspect of the quality assessment, the risk of bias was scored following the recommendations of the Cochrane Handbook for Systematic Reviews of Interventions 5.1.0 (http://handbook.cochrane.org). The judgment for each entry consisted of recording “yes” (low risk of bias), “no” (high risk of bias) or “unclear” (either lack of information or uncertainty over the potential for bias). We considered three out of the 6 domains in the Cochrane risk of bias tool as key domains (15). At study level, studies were judged to be at “low” risk of bias if there was adequate sequence generation, allocation concealment and blinding (operators and participants). If one or more criteria were not met, the study would be considered at “high” risk of bias. Study quality was rated on a scale from 0 (high risk of bias) to 9 (low risk of bias). In cohort studies, each item of the scale could be awarded one point. Only the item comparability could be awarded two points for a maximum of two adjusted confounders in the analysis. The studies were considered to be at high risk of bias in the case of a summarizing star score of ≤ 6, and at low risk of bias in the case of a score of > 6. Disagreements between the reviewers in relation to quality assessment were resolved by consensus or by consulting a third reviewer. Quality is based upon the number of stars reached.

- Eligibility criteria

Articles were included in this systematic review if they met the following inclusion criteria: systematic reviews, randomized clinical trials, controlled clinical trials, prospective and retrospective cohort studies and case series; only studies involving human individuals, aimed at showing efficacy of the all-on-four treatment concept, including ≥ 10 patients, with a minimum follow-up of three years, and reporting data related to treatment indication, surgical procedures, prosthetic protocols and complications (prosthetic and biological) associated to the all-on-four protocol.

Case reports, literature reviews, letters or comments to the editor, expert reports, *in vitro* and animal studies, as well as finite element studies or biomechanical tests were excluded from the present systematic review. Additionally, studies that assessed simultaneous implant placement with sinus lifting or regenerative procedures, zygomatic implants or the placement of more than four dental implants, without distal tilted implants following the all-on-four concept, as well as studies that did not evaluate immediate loading or applied loading more than one week after implant placement, were excluded.

- Data synthesis

The extracted data were stratified and expressed in chronological order according to publication date; data synthesis was based on evidence tables; and a descriptive summary was produced to obtain information related study variations (characteristics and results). If a study did not report raw data related to survival rates or implant failure, or prosthetic and biological complications, but did offer percentages regarding outcomes of interest, the summary was converted as required.

## Results

- Study screening

**Figure 1:**
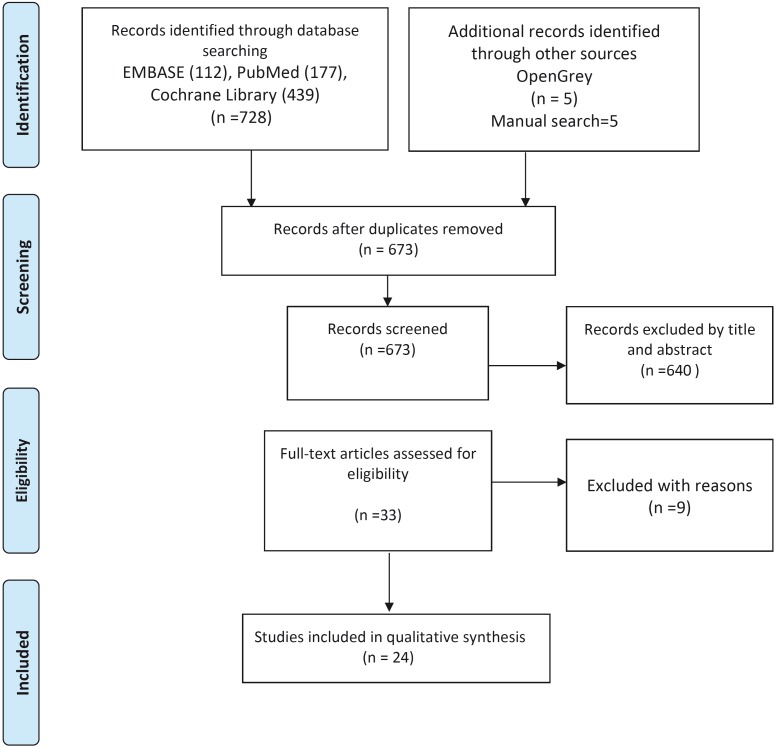
PRISMA flowchart of searching and selection process of titles during systematic review.

A total of 728 articles were obtained from the initial screening process: Medline - PubMed (n=177), EMBASE (n=112), the Cochrane Library (n=439) and OpenGrey (n=5). In addition, 5 titles were obtained through manual searching (references list and primary sources). Of these publications, 31 were identified as potentially eligible articles through screening by titles and abstracts. The full-text articles were subsequently obtained and thoroughly evaluated. As result, 24 articles fulfilled the inclusion criteria and were finally included in the present systematic review (Fig. 1). While information related to the excluded articles (with reasons) is presented in ( Table 1).

**Table 1:**
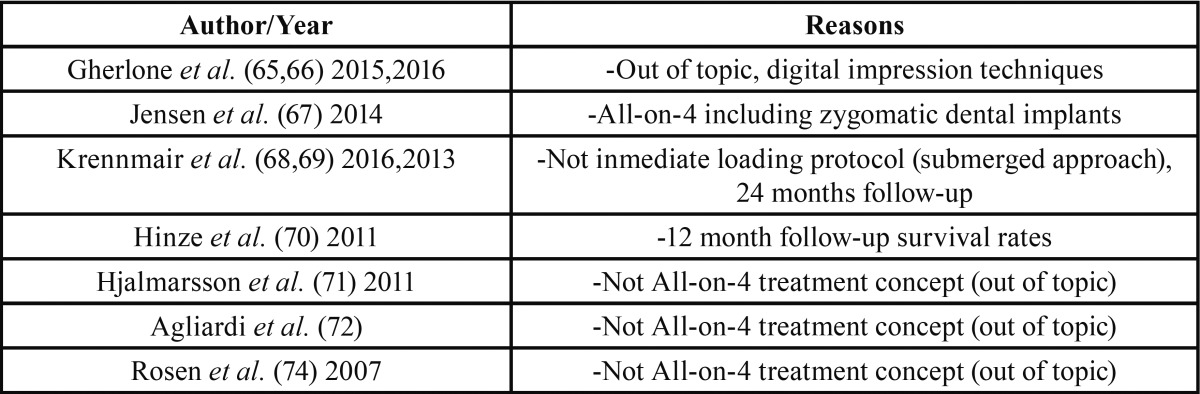
Articles excluded (with reasons) in the present systematic review.

- Included studies

Finally one randomized clinical trial was included (RCT)(16), 9 prospective studies (5 prospective single cohort studies (5,17-20) and 4 prospective case series (21-24)), and 14 retrospective studies (7 retrospective cohort studies (25-31) and 7 retrospective case series (32-38)).

- Methodological quality of the included studies

**Figure 2:**
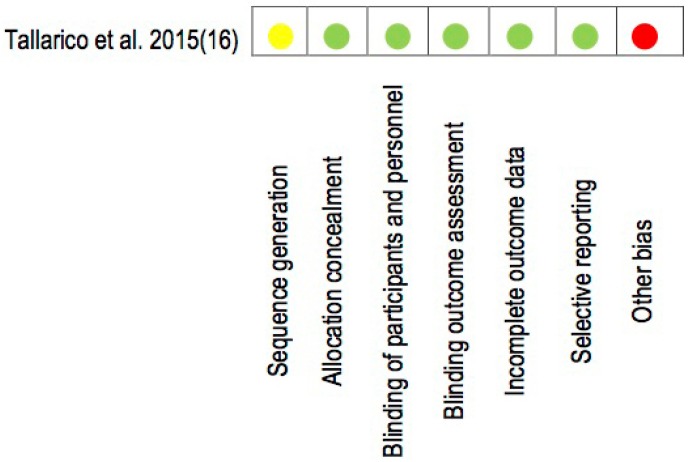
Cochrane Assesment Tool of Risk of Bias for RCT. Low risk of bias (green); high risk of bias (red), unclear risk of bias (yellow).

The 24 studies included in the present systematic review were prospective and retrospective observational studies, with only one experimental study (16) assessing the all-on-four treatment concept (Fig. 2). Substantial inter-rater agreement was obtained according to the Cohen kappa test, k = 0.78 (95% confidence interval 0.58- 0.86), based on the Landis & Koch scale (41).

Both longitudinal and retrospective cases series were methodologically treated as single cohort studies, due the fact that they evaluated only one type of treatment or exposure, without a comparator group.

In this manner, in 13 studies presenting a high risk of bias, the lack of methodological quality was related to incomplete follow-up, with attrition of clinical data, that could prove misleading on interpreting the results. All studies reported clear inclusion and exclusion criteria. The included studies were designed as single treatment studies. The quality assessment is summarized in  Table 2.

**Table 2:**
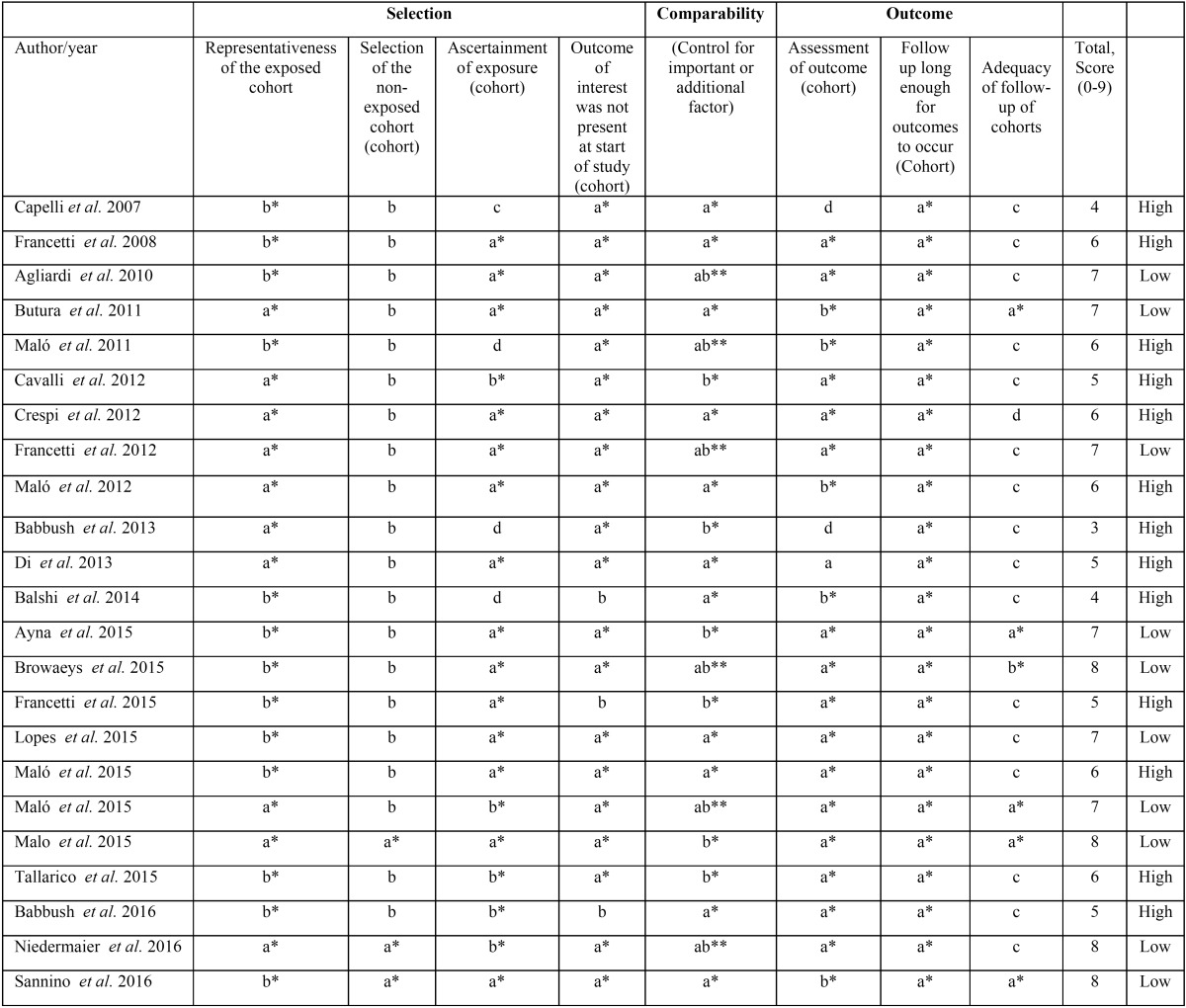
Methodological Quality Assessment of Non-Randomized Studies - Newcastle Ottawa Scale.

Only one study performed a sample size calculation (28). Calibration of the examiners was poorly described in the articles, and while some authors mentioned blinding of the evaluator, none described the way in which this was established. Only one study reported complete follow-up without sample attrition (16).

- Treatment indications

- Ridge condition, bone quality assessment and need for bone regeneration

The main treatment indication was an atrophic jaw or edentulous maxilla, with or without remnant hopeless tooth. Some studies considered as indication patient reluctance to undergo regenerative procedures such as sinus lift or bone grafts allowing implant placement in the posterior atrophic jaw (5,29,33).

Bone quality was assessed according to the criteria established by Lekholm & Zarb in 1985 (39) in some studies (6,22,26,35).

In the publication by Lopes *et al.* (24), the patients were classified according to the degree of surgical difficulty based on the residual ridge dimensions – difficulty being scored as low (residual ridge > 5 mm wide), moderate (irregular residual ridge 4-5 mm wide) or high (irregular residual ridge < 4 mm wide). In turn, Tallarico *et al.* (16), based on the Cawood & Howell classification, considering discrepancies in the degree of resorption as indication criteria.

- Indication of immediate loading and related insertion torque.

In relation to the indication of implant insertion to allow immediate rehabilitation, specific procedures were adopted for increasing primary stability of the implants during site preparation, such as the under-preparation of bone, dependent upon the bone strength observed during initial drilling (5,18,19,21,26). To allow immediate rehabilitation, the implants were inserted with a final torque of between 30-50 Ncm.

Moreover, two reports placed importance on jaw width and height in the interforaminal crest area as an indication on placing implants, these reports describe, a minimum required height of 6 mm, and at least >5 mm width and >8 mm height, respectively (21,22).

In the present systematic review, most authors considered the inclusion of healthy patients, compatible with an American Association of Anesthesiology (ASA) score of ASA I or II. However, some studies did not report this aspect related to patient surgical risk as an indication (16,18,19,26). Treatment indications were summarized in  Table 3.

**Table 3:**
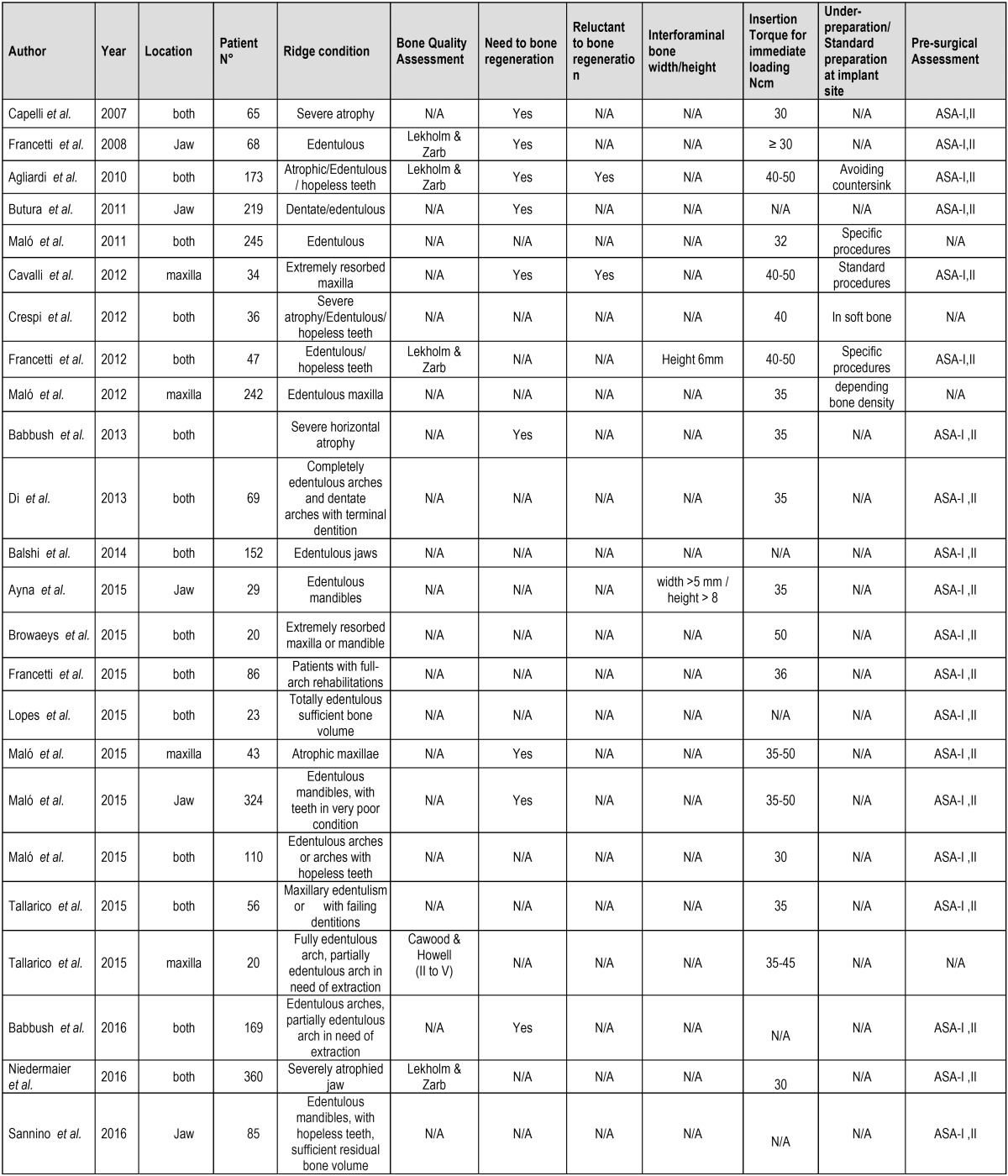
Treatment indications related to bone quality assessment and health conditions.

- Surgical procedures

- Sedation, incision and surgical anatomical reference

Prior to surgery, all authors used local anesthesia based on the infiltration technique, and some authors moreover used sedation with local anesthesia (5,17,18,25-27,29,32,37). On other hand, regarding the incision approach, a crestal incision was performed in both the maxilla and mandible, from the first molar to the same piece on the contralateral side.

Moreover, some authors perform a vertical distal incision in the maxilla to relieve the flap (5,19,25). However, when a guided surgical approach was programmed, the authors placed a computer-designed prosthetic splint with subsequent implant placement following a flapless technique (23,24,31).

Once incision and detachment were performed, and as a safety measure or as a way to orientate placement of the distal jaw implants, some authors made a window in the maxillary sinus, locating the mesial wall (5,20,25-27,37). The same procedure was used in the jaw until reaching the emergence of the mental nerve (21,26).

- Guided surgery

Of all the included studies, 17 used some kind of surgical guide to drill the implant bed in an attempt to secure optimal insertion with adequate inclination (16-24,26-31,37,38). Of the different types of guided surgery, the most widely used option was the Nobel Biocare System (16,21,29,38). Another commonly used tool was the all-on-four guide (21).

Some authors drilled a bed 2 mm in diameter on the midline, in the center of the ridge, to position implants both in the maxilla and mandible (23,26). In all studies describing hopeless or remnant teeth in the arch, these were removed before implant placement.

- Bone ridge regularization, distal implant angulation and insertion torque 

Regularization of the bone crest was performed if considered opportune by the operator in dentate patients undergoing tooth extraction in the same surgery (17,19,20,30,32-34). Regarding distal implant placement, we found similar inclinations among studies. However, Capelli *et al.* (2007) (25) reported that implants were placed angled a maximum of between 25° and 30°. Many authors placed the distal implants with an angulation of 30° (5,16,17,19,24,26,27,29,30,32).

Maló *et al.* (2015) (28,37) reported distal implant placement at 30° degrees, though in some cases they reached an inclination of up to 45° degrees, depending on the situation and anatomical location - in coincidence with other authors (18-20,22,31). The insertion torque of the implants described in the studies varied between 25 and 50 Ncm. Three authors described a torque of 30 Ncm (25,27,30,37), while 10 authors applied a torque of between 32 and 37 Ncm (18,20,22,26,34,38). In turn, other studies reported a torque between 40 and 50 Ncm (17,19,21,32,33), though few authors inserted the implants with a torque of 50 Ncm (5,23). However, no studies suggested the use of resonance frequency analysis to evaluate dental implant stability (e.g., Ostell).

- Implant length and diameter

The length and diameter of the implants - either axial or angled in maxilla or mandible - described in relation to this technique varied among the different studies analyzed. The shortest length, described by Malo *et al.* (2015) (27), was 7 mm, with a survival rate of 95.4% at three years, while the longest implant length was 18 mm (5,18,32). The average length used in the studies was 10 mm. In turn, the smallest reported diameter was 3.3 mm (16,26), with a maximum of 5 mm, described by Niedermaier *et al.* (30). Additional data are depicted in  Table 4.

**Table 4:**
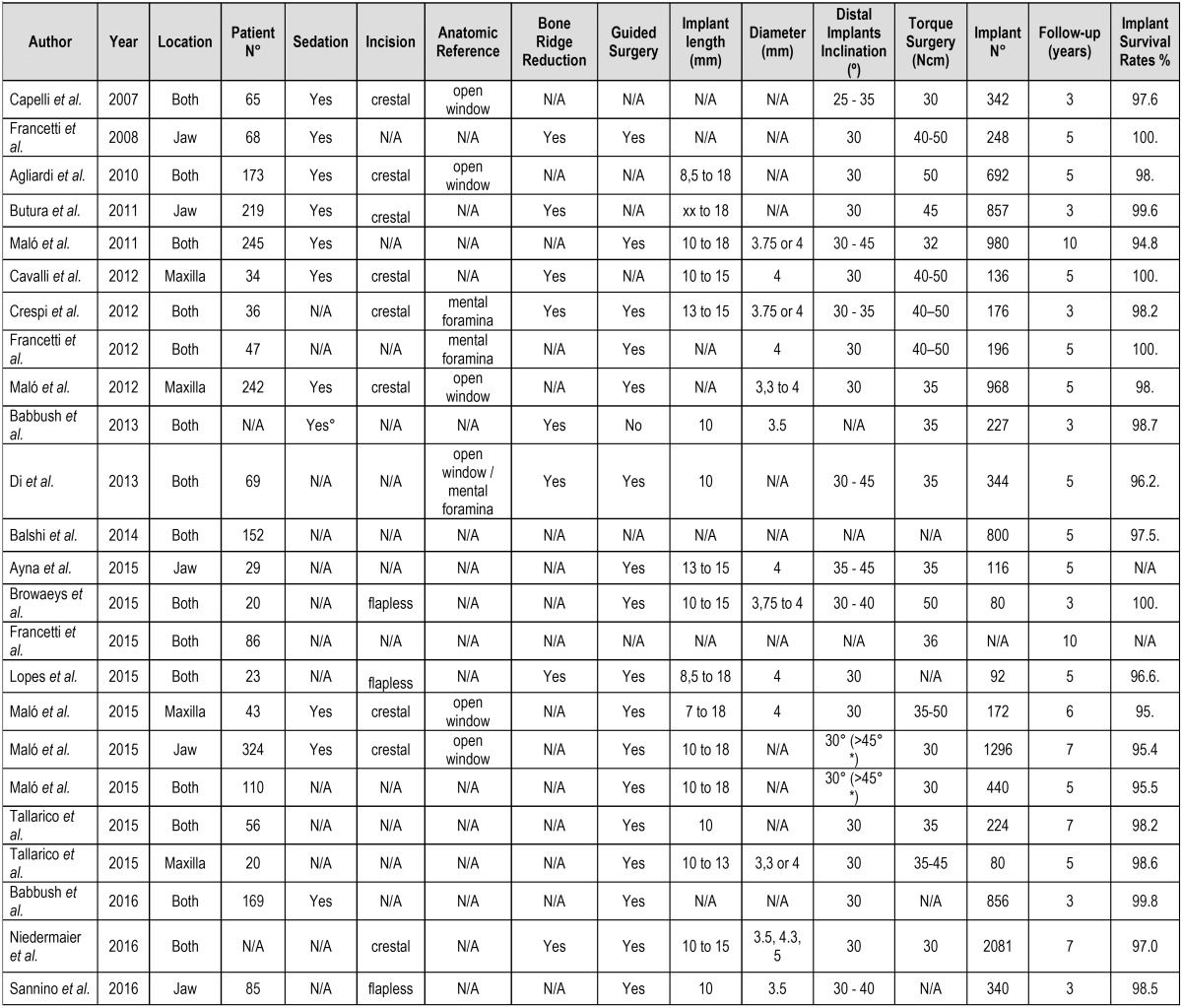
Surgical procedures.

Prosthetic protocols

- Immediate and definitive loading

Immediate loading protocols were used after 48 hours in 6 studies (17,21,23,25,33,36), after 24 hours in other studies (22,34,37), and on the same day a minimum of two hours to a maximum of 8 hours after surgery (5,18,26,29,31,32). Only three studies did not offer information on this aspect. Fourteen studies performed definitive prosthetic loading after between 4-6 months. In contrast, only one study performed definitive loading after two months (25), while two studies performed permanent loading three months after provisional loading (29,30).

- Provisional prosthetic material 

Most of the reports in the present review showed a preference for acrylic resin materials with different nomenclatures (acrylic resin, high density acrylic, resin based), and in some cases these prostheses were reinforced with a titanium or metal framework (25,31,38) or with titanium cylinders (18,26-28,37). Only 5 studies indicated the number of teeth included in the prostheses (5,17,18,21,23) - the number being 10 to 12 teeth in some studies, without cantilever.

- Definitive prosthetic material

The definitive prostheses were fabricated using CAD-CAM in some studies, or were made with metal-ceramic materials, reinforced with titanium frameworks. Denture extension mainly comprised in 12 teeth, and one study reported the use of zirconia crowns, while other reports described the use of acrylic resin prostheses with a titanium framework and acrylic-resin prosthetic teeth, elaborated with high density acrylic material and titanium cylinders.

- Abutment type and prosthetic screw tightness 

In relation to prosthetic abutment inclination, most of the studies described the use of both tilted and straight types inclined between 17° to 35°, being indicated to compensate the lack of parallelism between implants. Straight and 17° angulated multiunit abutments were frequently used on anterior implants, and 30° angulated abutments were most commonly used on distal implants, as reported by some authors (24,25,33). Data referred to prosthetic screw tightening using a torque controller were provided by a few studies - the applied forces being in the order of 10-20 Ncm (5,17,25,32,33).

- Occlusion control and prosthetic settlement assessment

Many studies treated interferences in excursive dynamic movements through the establishment of centric and lateral contacts within the inter-canine zone, in attempting to secure mutually protected occlusion. Only a few articles failed to provide information in this regard (24,25,34). In addition, mutually protected occlusion with anterior guidance or balanced occlusion was used in cases of opposing natural dentition, or an FDP and complete removable denture, respectively, as described by Tallarico *et al.* (2016) (38). Other approaches were also described by some authors, considering as static occlusion that comprising central contacts established on all masticatory units but the cantilevers for the first three months (30).

However, two studies described particular methods in comparison with other reports. In effect, Ayna *et al.* (2015) (22) described the use of pressure sensitive film using a software application called Appendant, and Browaeys *et al.* (2015) indicated that evaluation was carried out by a prosthodontist (23).

Prosthetic settlement and implant placement was checked from panoramic and periapical radiographs taken using the parallel projection technique to guide fitting of the prostheses and abutments, though few studies described this procedure (18,27,34,36). Additional data regarding prosthetic protocols are depicted in  Table 5,  Table 5 continue.

**Table 5:**
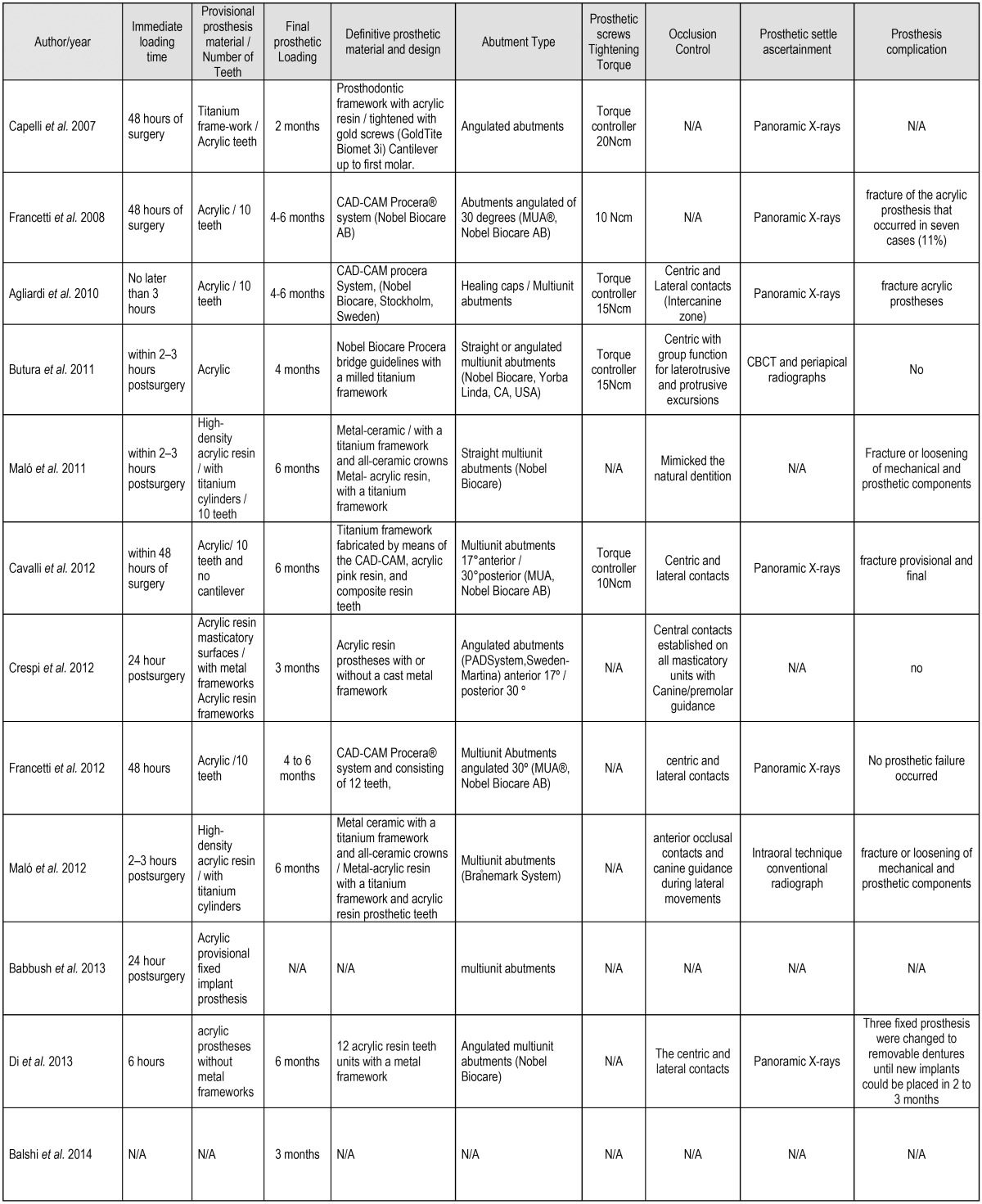
Prosthetic protocols.

**Table 5 continue:**
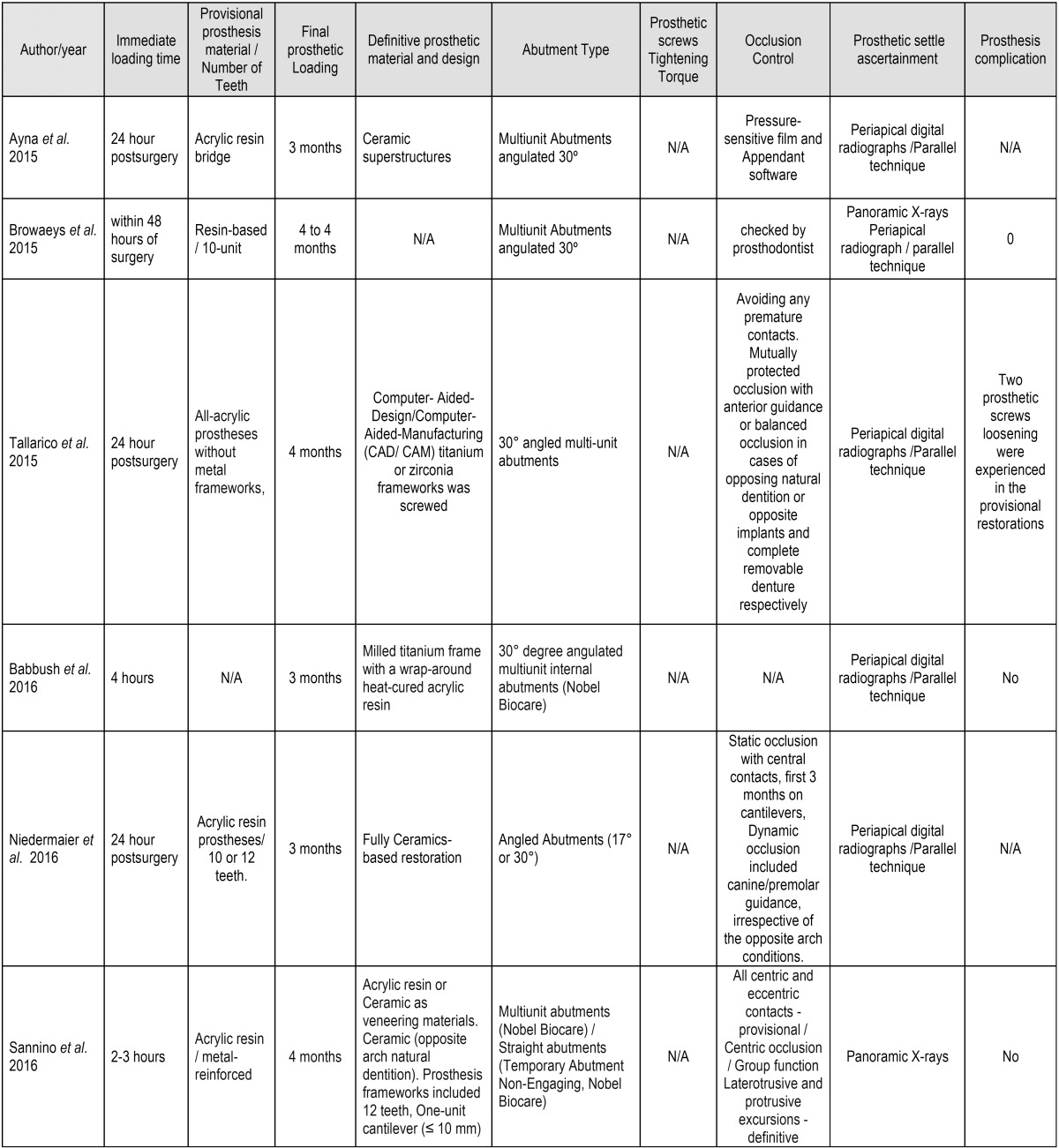
Prosthetic protocols.

Patient satisfaction was assessed by means of a questionnaire. All patients were satisfied with the phonetic, esthetic, psychological and functional results once treatment was completed (17,25,31). Only one study reported satisfaction assessed on the basis of percentages using a visual analog scale (VAS) for masticatory, phonetic and esthetic outcome (31).

 A high degree of patient satisfaction was reported in relation to this clinical procedure (25,33). Patient satisfaction with the all-on-four treatment concept was very high (rated as excellent by 95.6% of the patients)(20).

- Mechanical complications

Some authors assessed mechanical complications such as fractures or loosening of prosthetic components. The most frequent prosthetic complication was fracture of the acrylic prostheses, which occurred in 9 clinical studies (further details are provided in  Table 5). These problems were resolved by repairing the prostheses, adjusting the occlusion, and manufacturing and using an occlusal nightguard (36). See  Table 5.

These situations were resolved by retightening the screws, controlling the occlusion and advising the patients to not overload the prostheses (i.e., avoiding food that could require significant chewing effort) (18). Of all the technical and prosthetic complications, the detachment of an element of the definitive prosthesis was the most frequent problem (recorded in 23.2% of the patients) (24,36).

Five studies reported no prosthetic complications (19,21,29,31,32). Most authors reported that such technical and mechanical complications do not affect the survival rate of either implants or prostheses.

- Biological complications

The 24 articles yielded information on biological complications related to 11,743 implants placed. Of these implants, 134 failed during the first year, 9 implants failed before two years, and 31 implants failed during an interval of 3-10 years. In total, 175 implants were unsuccessful. (Data not shown).

The lowest reported success rate was 94.8% in 245 patients at 10 years (18). The most frequent complication was the loss of at least one implant. Only two studies reported a cumulative success rate of 100% at implant level (17,33). In turn, the second most frequent complication was the development of peri-implantitis after two years (16,17,27,33,36,38), and some studies reported cases of mucositis (33,36).

These complications were reported without precise definitions or detailed comments on the topic. Other authors (18,24) described cases of infection of at least one implant. There were no permanent lesions, though Francetti *et al.* (2008) (17) reported a case of paresthesia that resolved within 6 months.

The minimum survival rate at 36 months was 97.6% (25). Curiously, Browaeys *et al.* describe a survival rate of 100% in 80 implants (40). On the other hand, Malo *et al.* (18) described long-term survival rates of around 95% in 172 implants in a clinical study with a follow-up ranging from 5-10 years. These were the only authors to report a success rate of 100% in 176 placed implants.

## Discussion

Principal findings

Settlement misfit in removable complete dentures can cause soreness and patient discomfort, and is a consequence of severe bone resorption/atrophy of the jaws (42), with a direct impact upon patient quality of life (43). The magnitude of these changes is important for decision-making and comprehensive treatment planning (44), and has a considerable impact on tooth replacement therapy, particularly when implant-supported restorations are planned (45).

The all-on-four treatment concept arises as an attempt to allow treatment with affordable time and cost through immediate implant-supported restorations, providing relatively straightforward and predictable treatment in edentulous patients with atrophic jaws. The outcome is favorable in terms of quality of life (9), when compared with the traditional 3-6 months during which the fixtures are protected from premature loading (46,47), requiring second surgery to expose them and connect the transmucosal components, and increasing the time and cost of treatment, as well as patient morbidity.

The present systematic review sheds light upon the therapeutic indications, surgical procedures, prosthetic protocols, patient satisfaction and main complications (both technical and biological) associated to the all-on-four treatment concept, with the aim of clarifying and supporting application of the protocol in different clinical situations, and improving understanding and decision making in everyday clinical practice.

Lekholm & Zarb classification was the method to evaluate bone quality most frequently used in by studies included in the present review (39). However, bone quality was only assessed during the implant drilling; no additional data, such as minimum bone quantity available, that may help in clinical decision making was provided by the studies. Only the study of Lopes *et al.* (24) describes that patients were classified according to surgical difficulty based on the residual ridge dimensions as follows – difficulty being scored as low (residual ridge > 5 mm wide), moderate (irregular residual ridge 4-5 mm wide) or high (irregular residual ridge < 4 mm wide).

However, another classification has been described by Jensen in 2014 (48) and may serve as a complement, helping during treatment indications, in patients receiving immediate full-arch implant retained prostheses following the all-on-four concept.

Mention should be made of the study by Tallarico *et al.* (16), which describes the Cawood & Howell classification as indication criterion, considering discrepancies in the degree of resorption. The study indicates that in patients corresponding to Cawood & Howell class IV, V and VI, the all-on-four treatment concept seems to be a safe, effective and efficient surgical-prosthetic protocol applied to both jaws, avoiding technique-sensitive augmentation procedures (49,50).

Moreover, regarding the indication to perform immediate loading in relation to insertion torque, the present review found the implants to be inserted with a final torque between 30-50 Ncm. The insertion torque is frequently enhanced through implant site under-drilling by avoiding the countersink to maximize implant stability (5). This approach is biologically plausible due to the fact that mechanical stimulation around a recently placed implant positively modulates the release of bone mediators around immediately loaded implants.

Malo *et al.* (2011) described the protocol for the insertion of implants following standard procedures, except that under-preparation was used to achieve an insertion torque of at least 35 Ncm before final seating of the implant. The authors showed this to be typically done by full drill depth with a 2-mm twist drill followed by step drills of 2.4/2.8 mm and 3.2/3.6 mm (depending on bone density). In cases of high bone density, 3.8/4.2 mm step drills were used only in cortical bone. The implant neck was aimed to be positioned at bone level, and bicortical anchorage was established whenever possible (26).

However, some authors indicate that loading dental implants indiscriminately and immediately is not safe because of potentially unfavorable stress distribution and a negative cellular response under such high stress during early healing, when the implants are not splinted, as in unsplinted implants in dental overdentures or partial fixed dental prostheses (52). Moreover, insertion of implants with high torque following an under-drilling protocol - commonly used for immediate loading - may reduce crestal bone-to-implant contact in the early healing stages, as recently demonstrated in a pre-clinical study. However, more prospective clinical evidence is needed to confirm this (53).

In relation to the surgical procedures used, many authors administer local anesthesia based on the infiltration technique, though there has also been a description of sedation (via the oral or intravenous route) with local anesthesia. It is important to consider that sedation with benzodiazepines during surgical procedures such as third molar extractions is associated to anesthetic complications in adolescents, mainly among those administered diazepam, with a 50% increase in the risk of adverse complications (54). On the other hand, no study has reported complications related to the sedation procedure during implant surgery following the all-on-four treatment concept.

In our opinion, this is a relevant topic, since Flanagan (2004) indicated that benzodiazepines such as triazolam are contraindicated in pregnant or nursing patients, and well as in individuals who consume alcohol or are under treatment with macrolide antibiotics, certain protease inhibitors, psychotropic agents, ketoconazole, itraconazole, nefazodone, or other medications that impair oxidative metabolism mediated by the cytochrome P450 3A (CYP 3A isoenzyme) metabolic pathway. It is suggested that triazolam should be used with caution in patients who consume grapefruit juice or receive cyclosporines and other drugs such as calcium channel blockers including nifedipine, verapamil, and diltiazem (55). Sedation is an interesting topic for future studies.

Regarding the extent of the surgical incision, it has been performed from the first molar to the same piece on the contralateral side, both in maxilla and mandible. On the other hand, some authors prefer to perform a vertical distal incision to relieve the flap (5,19,25), allowing improved access to the surgical site. After flap reflection and detection of the mental foramina, the length of the mental nerve loop and the shape of the bone were assessed using an atraumatic instrument, in order to determine the ideal angulation of the posterior implants.

However, nowadays the trend it is to minimize patient morbidity. In this sense, some authors have introduced the concept of flapless surgery through the use of prefabricated and customized guides based on stereolithographic casts, in an attempt to enhance accuracy during surgery and safely avoid the need for critical anatomical repairs (56).

Some authors report the use of guided surgery to obtain optimal insertion with adequate angle inclination – this being an affordable choice for full-arch fixed restorations with immediate loading. However, associated complications such as implant loss, prosthetic or surgical guide fractures, and low primary stability are often observed, and there is a learning curve for ensuring treatment success, as recently reported by a systematic review (57).

Regarding implant inclination, the reported angulations vary between 30 to 45 degrees, although this depends on the anatomical location (18-20,22,31). The use of tilted implants to support fixed partial and full-arch prostheses for the rehabilitation of edentulous jaws can be considered a predictable technique, with an excellent prognosis over the short and middle term (58), though it has been suggested that differences in angulation of dental implants might not affect implant survival or marginal bone loss (59).

Since primary stability plays a critical role in osseointegration, a greater insertion torque is more desirable, and shows better effects if the implants are splinted through a full-arch restoration with immediate loading than when single crowns are considered, where the effects prove risky for implant survival (60).

The insertion torques reported are heterogeneous, there are reports indicating that around 25 at 50 N/cm were applied, moreover the use of ISQ values to assess implant stability were not described between studies. These data do not seem to exert an effect upon dental implant survival (61). However, excess insertion torque may possibly cause wearing on the implant surface, generating a foreign body reaction due to titanium debris and ions released from the surface (62).

The present review indicates that all compromised teeth were extracted, and sockets were carefully debrided, before placement of the implants (5). Subsequently, the ridge crest was trimmed to remove any sharp edges, as reported by Ping Di *et al.* (20). This approach has been optimized through the use of stereolithographic models, as commented above (56).

However, it is important to consider the reasons for tooth extraction, since previous reports point to a critical role of periodontitis as a contributor to mucositis and peri-implantitis, which seems to be related to implant loss (63).

The most frequent prosthetic complication was fracture of the acrylic prostheses - such situations being resolved through relining and occlusion adjustment, with the use of an occlusal nightguard (36) - as well as prosthetic screw losses, which are resolved by retightening the screws, controlling occlusion and advising the patients to not overload the prostheses (18). With regard to the technical and prosthetic complications, the detachment of an element of the definitive prosthesis was the most frequent problem (recorded in 23.2% of the patients) according to two studies (24,36). These observations are consistent with the results of a recent study on tooth fractures in fixed full-arch implant-supported acrylic resin prostheses. The authors concluded that such fractures are a common complication, and that several factors are more directly associated to the need for mechanical maintenance (64).

These authors also reported that fractures were frequently observed when the opposing arch included only natural teeth, and were more common than in the case of full dentures or implant-supported overdentures, due to the greater force these patients can apply, as well as to the abrasiveness of the natural enamel or the fixed ceramic prostheses that could form part of the arch. In cases with full arch implant supported prostheses in both arches, a high incidence of fractures has been described, which could be due to reduced proprioception (64).

The quality of the evidence in this interesting topic in implant dentistry requires more clinical trials, with a good design and sample size estimation, and adequate follow-up without sample attrition, in order to try to answer the questions related to the advantages of this treatment referred mainly to implant survival rates and biological complications that are poorly described in the available literature. Consideration is also required of the patient-related outcomes (Proms) to compliment surrogate clinical outcomes, because in the present systematic review only three studies (17,25,31) assessed patient satisfaction using questionnaires and visual analog scales.

## Conclusions

The all-on-four treatment concept offers a predictable way to treat the atrophic jaw in patients that do not prefer regenerative procedures, which increase morbidity and the treatment fees. The results obtained indicate a survival rate for more than 24 months of 99.8%.

The open window technique to ascertain the anterior wall of the sinus allows adequate implant insertion, and in the jaw to denudate the emergence of the mental foramina. Under-preparation of the implant bed was performed to obtain better primary stability, avoiding countersink in cortical bone.

This protocol may be performed through guided surgery following the flapless approach or using the open flap approach with a metallic surgical guide to enhance accuracy and ensure adequate positioning and inclination of distal implants.

Prosthetic complications such as acrylic fracture or the detachment of prosthetic parts were frequently reported. Moreover, acrylic resin materials, with or without reinforced titanium or metal structure, were preferentially used in definitive prostheses. The main biological complications (e.g., peri-implantitis) were reported in few patients after a mean follow-up of two years.

However, current evidence is limited due the scarcity of information referred to methodological quality, a lack of adequate follow-up, and sample attrition. Adequate definition of the success / survival criteria is thus necessary, due the high prevalence of peri-implant diseases.
